# Circulating tumor DNA for prognosis assessment and postoperative management after curative‐intent resection of colorectal liver metastases

**DOI:** 10.1002/ijc.33924

**Published:** 2022-01-19

**Authors:** Thomas Reinert, Lena Marie Skindhøj Petersen, Tenna Vesterman Henriksen, Marie Øbo Larsen, Mads Heilskov Rasmussen, Amanda Frydendahl Boll Johansen, Nadia Øgaard, Michael Knudsen, Iver Nordentoft, Søren Vang, Søren Rasmus Palmelund Krag, Anders Riegels Knudsen, Frank Viborg Mortensen, Claus Lindbjerg Andersen

**Affiliations:** ^1^ Department of Molecular Medicine Aarhus University Hospital Aarhus Denmark; ^2^ Department of Surgery Aarhus University Hospital Aarhus Denmark; ^3^ Department of Pathology Aarhus University Hospital Aarhus Denmark

**Keywords:** circulating tumor DNA, colorectal cancer liver metastases, droplet digital PCR, minimal residual disease, recurrence surveillance

## Abstract

The recurrence rate of colorectal liver metastases (CRLM) patients treated with curative intent is above 50%. Standard of care surveillance includes intensive computed tomographic (CT) imaging as well as carcinoembryonic antigen (CEA) measurements. Nonetheless, relapse detection often happens too late to resume curative treatment. This longitudinal cohort study enrolled 115 patients with plasma samples (N = 439) prospectively collected before surgery, postoperatively at day 30 and every third month for up to 3 years. Droplet digital PCR (ddPCR) was used to monitor serial plasma samples for somatic mutations. Assessment of ctDNA status either immediately after surgery, or serially during surveillance, stratified the patients into groups of high and low recurrence risk (hazard ratio [HR], 7.6; 95% CI, 3.0‐19.7; *P* < .0001; and HR, 4.3; 95% CI, 2.3‐8.1; *P* < .0001, respectively). The positive predictive value (PPV) of ctDNA was 100% in all postoperative analyses. In multivariable analyses, postoperative ctDNA status was the only consistently significant risk marker associated with relapse (*P* < .0001). Indeterminate CT findings were observed for 30.8% (21/68) of patients. All patients (9/21) that were ctDNA positive at the time of the indeterminate CT scan later relapsed, contrasting 42.6% (5/12) of those ctDNA negative (*P* = .0046). Recurrence diagnoses in patients with indeterminate CT findings were delayed (median 2.8 months, *P* < .0001). ctDNA status is strongly associated with detection of minimal residual disease and early detection of relapse. Furthermore, ctDNA status can potentially contribute to clinical decision‐making in case of indeterminate CT findings, reducing time‐to‐intervention.

## Graphical abstract

What's new?

Current surveillance methods after surgery for colorectal cancer liver metastasis are too often inadequate, and the cancer recurs after resection in more than half of patients. Here, the authors assess the predictive value of ctDNA analysis. They collected plasma from 115 patients before surgery, 30 days after surgery and then quarterly for up to 3 years. For each patient, one tumor‐associated mutation was assessed in the plasma. Postoperative detection of tumor ctDNA was highly predictive of relapse. Analysis of ctDNA could complement other surveillance methods to guide treatment decisions.

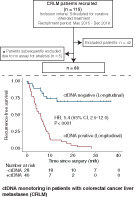

**Abbreviations**
CEA: carcinoembryonic antigen
cfDNA: cell‐free DNA
CI: confidence interval
CRC: colorectal cancer
CRLM: colorectal liver metastases
CT: computed tomography
ctDNA: circulating tumor DNA
ddPCR: droplet digital PCR
FF: fresh frozen
FFPE: formalin‐fixed paraffin‐embedded
HR: hazard ratio
LOD: limit of detection
N stage: nodal stage
NGS: next generation sequencing
OR: odds ratio
PCR: polymerase chain reaction
PON: panel of normal
RFS: recurrence‐free survival
UMI: unique molecular identifier
VAF: variant allele frequency

## 1 INTRODUCTION

Colorectal cancer (CRC) is the third most common type of cancer worldwide.^1^ Liver involvement is the primary cause of death for CRC patients with approximately half of the patients developing colorectal liver metastases (CRLM).^2^ The last decade has seen an increasingly aggressive approach to management of CRLM, often with the intention to cure.^3^ Nevertheless, the recurrence rate after curative intent treatment for CRLM remains high, with nearly 50% recurring within 2 years^4^, ^5^, ^6^ and a 5‐year disease‐free survival of only 27.9%.^7^ Most CRLM surveillance programs involve frequent computed tomographic (CT) scans.^8^, ^9^ A common challenge related to this, is indeterminate CT findings in the liver and lungs.^10^, ^11^ Often, they represent benign lesions, but results in additional investigations and increased patient unease.^11^ In other instances, the indeterminate findings represent malignant lesions, but the requirement for further investigations before the final diagnosis often leads to delayed intervention.^11^, ^12^ Currently, there are no validated biomarkers of patient recurrence risk that could inform and personalize the use of chemotherapy and help guide and resolve the issues related to CT‐imaging based surveillance. Detection of circulating tumor DNA (ctDNA) by the use of tumor‐specific DNA mutations is an emerging biomarker, which in the setting of localized CRC has been shown to have potential to change the fields of postoperative prognostication and surveillance.^13^, ^14^, ^15^, ^16^, ^17^, ^18^, ^19^ Recent studies have indicated a similar potential for clinical application also in the setting of CRLM.^20^, ^21^ However, further prospective studies confirming and expanding on these findings are needed. Here, we report findings from a prospective and observational biomarker study in the setting of CRLM, aiming to assess the clinical value of serial ctDNA analysis for postoperative prognostication and guidance of CT‐imaging in patients treated with curative intent.

## 2 METHODS

### 2.1 Patients and sample acquisition

**FIGURE 1:**
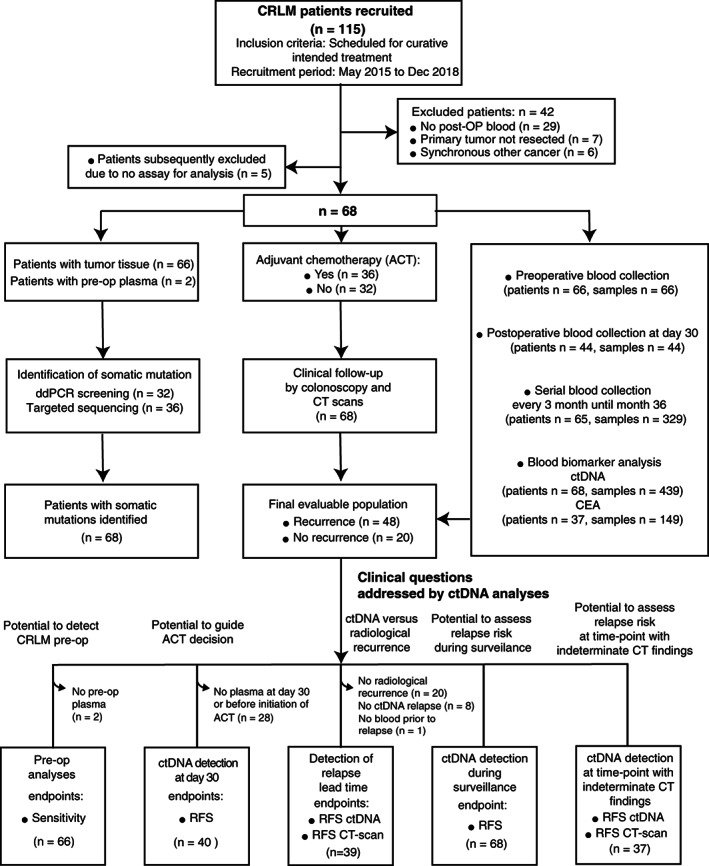
Patient enrollment, sample collection and definition of the patient subgroups used to address the defined clinical questions. N = 29: No post‐OP blood (The patients did not provide any blood samples after CRLM resection), N = 7: Primary tumor not resected (the scheduled resection of the primary cancer was canceled), N = 6: Synchronous other cancer, N = 5: No assay for analysis. ACT, adjuvant chemotherapy; ctDNA, circulating tumor DNA; CT, computed tomography; post‐op, postoperative; RFS, recurrence free survival

This prospective observational study enrolled patients eligible to curative intended treatment for CRLM at the Department of Surgery, Aarhus University Hospital (Figure 1) from 1 May 2015 to 31 December 2018. Prior to CRLM resection the patients received standard of care diagnostic work‐up, including CT scan of the thorax and abdomen. The primary tumor either had been previously radically resected (R0) or was deemed to be radically resectable (R0) by a classical, combined or liver‐first surgical strategy. The inclusion criteria of the study were: (a) No extrahepatic disease, (b) Eligibility for a R0 resection leaving at least 25% of functioning liver parenchyma and (c) A performance status allowing liver surgery. Tissue was collected from the resected CRLM specimen. Blood samples were collected prior to liver resection (up to 14 days prior), and at postoperative day 30 (ie, sample drawn up to 14 days before or after day 30), and then at every third month until death, patient withdrawal from the study or month 36, whichever came first. Common reasons for why some patients abstained from providing or were unable to provide post‐OP blood samples include: (a) need for help with transportation to get to the hospital making it inconvenient with frequent blood draws, (b) living far from the hospital making it inconvenient with frequent blood draws, (c) surgical complications, (d) difficulties recovering from surgery, (e) adverse effects of chemotherapy and (f) increased anxiety related to the disease and intervention. Clinicopathological information was collected for all patients (Tables 1 and Table S1). All patients received treatment and follow‐up in compliance with the National Guidelines defined by the Danish Liver Cancer and Biliary Cancer Group (DLGCG). The ctDNA analyses were performed retrospectively and blinded to sample order and patient outcome at the Department of Molecular Medicine, Aarhus University Hospital. Neither treating clinicians nor patients were informed about the ctDNA results.

**TABLE 1:** Patient and clinical characteristics

Patients, n	68
Age (years), median (range)	67.7, (45.1‐89.4)
Gender, n (%)	
Female	27 (39.7)
Male	41 (60.3)
Adj. therapy, n (%)^a^	37 (54.4)
Location of primary tumor, n (%)	
Colon	44 (64.7)
Rectum	24 (35.3)
Primary tumor N stage, n (%)	
N0	18 (26.5)
N+	44 (64.7)
NA^b^	6 (8.8)
Synchronous liver metastases, n (%)^c^	
No	37 (54.4)
Yes	30 (44.1)
NA	1 (1.5)
Number of liver metastases, n (%)	
Single	29 (43.6)
Multi	36 (52.9)
NA	3 (4.4)
Diameter of largest liver metastasis, n (%)	
≤3 cm	24 (35.3)
>3 cm	26 (38.2)
NA	18 (26.5)
KRAS mutations status, n (%)	
Wildtype	36 (52.9)
Mutated	30 (44.1)
NA	2 (2.9)
Patients with indetermined CT findings, n (%)^d^	21(30.9)
Relapse, n (%)	48 (70.6)
Relapse site (first), n (%)	
Oligo—liver	29 (42.6)
Oligo—lung	13 (19.1)
Multiple—liver and lung	4 (5.9)
Oligo—other	2 (2.9)
Lung relapse diameter (mm), mean (SD)	8.1 (±2.7)

^a^ Only after both CRC and liver resection.

^b^ Not available.

^c^ Synchronous liver metastases: synchronous diagnosis of primary colorectal tumor and metastatic disease.

^d^ Only the first nonspecific finding from each patient.

### 2.2 DNA extraction

CRLM tissue was collected, either as fresh frozen or as formalin fixed and paraffin embedded tissue (FFPE). Prior to DNA extraction DNA the cancer cell content in each biopsy was assessed by evaluation of hematoxylin and eosin stained sections cut before and after those used for extraction. The median cancer cell content was 50%. From fresh frozen tissue DNA was extracted using the Puregene DNA purification kit (Gentra Systems) and from FFPE tissue using the QIAamp DNA FFPE tissue kit (Qiagen). From all patients constitutional DNA was extracted from peripheral blood leukocytes using QIAsymphony DNA Midi Kit (Qiagen).

### 2.3 Blood collection and plasma isolation

Blood samples were collected in K2‐EDTA 10 mL tubes (Becton Dickinson) and processed to plasma and buffy coat within 2 hours of collection by double centrifugation at room temperature. First the blood sample was centrifuged for 10 minutes at 3000*g* and then the plasma was centrifuged for 10 minutes at 3000*g*. Plasma was aliquoted into 5 mL cryotubes and stored at −80°C.

### 2.4 Tumor screening for mutations in KRAS codon 12 and 13 and BRAF codon 600

To identify a tumor specific clonal mutation to be used as a marker of tumor DNA in droplet digital PCR (ddPCR) based plasma cell free DNA (cfDNA) analyses, the liver metastases were initially screened by the ddPCR KRAS G12/G13 Screening Kit (Biorad). KRAS positive patients were subsequently profiled by individual KRAS mutation assays to identify the specific mutations. KRAS negative samples were screened with a BRAF V600E mutation assay (Table S2).

### 2.5 Tumor mutational profiling by Memorial Sloan Kettering‐Integrated Mutation Profiling of Actionable Cancer Targets

For a subset of patients (N = 17) tumor mutational profiling was performed by targeted sequencing using the Memorial Sloan Kettering‐Integrated Mutation Profiling of Actionable Cancer Targets (MSK‐IMPACT) panel with the aim to identify a clonal mutation for use in plasma ddPCR (Table S2). The MSK IMPACT panel targets 468 cancer genes and includes probes targeting single‐nucleotide‐polymorphisms to enable the concordance between matched samples to be assessed.^22^ In brief, tumor and germline DNA was sheared using the Covaris E200 instrument (Covaris, Woburn, Massachusetts). Libraries from fragmented tumor and matched normal DNAs were prepared using the KAPA Hyper Library Preparation Kit (KAPA Biosystems). Libraries were indexed prior to pooling and subsequently paired‐end sequenced using Illumina NextSeq Medium v2, 300 bp flow cells. Adapters were trimmed using Trim Galore version 0.4.1 (Babraham Bioinformatics). Sequences were mapped to the hg19 reference genome using BWA‐MEM (Burrows‐Wheeler Aligner) software version 0.7.5a. Picard MarkDuplicates version 1.141 was used to remove duplicates and inspect the alignment. GATK IndelRealigner version 3.5 was applied for refinement of mapping in INDEL areas. GATK BaseRecalibrator was applied to identify and modify systematic errors in base quality scores. Somatic SNVs and INDELs were called using GATK MuTect2 version 3.5. The sequencing coverage and quality statistics for each sample are summarized in Table S3.

### 2.6 Mutational profiling by targeted duplex sequencing

Another subset of tissue samples (N = 23) and two preoperative plasma samples from patients, where no liver metastasis tissue was available were analyzed by ultra‐deep targeted duplex sequencing to identify the clonal mutation to be used in plasma ddPCR (Table S3). Duplex sequencing performed as described by Kennedy et al with some minor adjustments.^23^ In brief, libraries were generated using KAPA Hyper Library Preparation Kit (KAPA Biosystems). Prior to library preparation, DNA from tumor (N = 25) and buffy coat was fragmented by sonication to an average size of 300 to 400 bp. DNA from plasma was not fragmented (N = 2). DNA was end‐repaired and A‐tailed according to standard protocol. This was followed by ligation of adapters with an incubation time of 30 minutes. Adapters were synthesized in‐house following the protocol by Kennedy et al and were designed to contain a random double stranded unique molecular identifier (UMI) of 6 and 12 nucleotides mixed in equal quantities. The adapter‐ligated libraries were purified using a 1.4× SPRI purification with Ampure XP beads. Hereafter, a sample specific index was added to the libraries by PCR, followed by a 1.0× SPRI purification. During PCR, all reactions were split in four to reduce PCR amplification bias. After library generation, the genomic fragments of interest were selected using a custom‐made capture panel targeting 12 genes recurrently mutated in CRC (Table S4). The panel included probes targeting single‐nucleotide‐polymorphisms (N > 18), which enabled the concordance between matched samples to be tested. Paired end NGS data were processed using a custom pipeline. In brief, UMIs were extracted and the reads mapped to hg19 using bwa mem (v0.7.17) Potential PCR errors within the UMIs were repaired using umi_tools group (v1.0.0). A consensus data set was constructed by collapsing all UMI families. Consensus reads were realigned using GATK RealignerTargetCreator and IndelRealigner (v3.8), and a pileup format generated using pysamstats (v1.1.2). A panel of normal (PON) samples, consisting of cfDNA from 75 normal healthy, were sequenced by Duplex sequencing and used to establish a background error model and base‐specific dispersion factors. Using these, variants were called using Shearwater algorithm of deepSNV R package.^24^ For variant calling the shearwater algorithm computes a Bayes classifier based on a beta‐binomial model. A Bayes factor‐cutoff = 0.05 was used to call mutations.

### 2.7 Cell‐free DNA extraction and quantification

DNA was extracted from 8 mL plasma by Qiasymphony DSP Circulating DNA Kit Cat#937556 (Qiagen). Assessment of plasma DNA purification efficiency, lymphocyte DNA contamination and quantification of the cfDNA content was carried out using ddPCR as previously described.^17^ The primer and probe sequences of the used ddPCR assays are provided in Table S5.

### 2.8 Droplet digital PCR assays for quantification of ctDNA

The selection of mutations for ctDNA analysis was restricted to mutations in the genes APC, BRAF, KRAS, NRAS, PIK3CA and TP53. Mutations with variant allele frequencies below 25% of the histology estimated tumor fraction were judged to be subclonal, and not selected for ctDNA analysis. For each patient matched white blood cell DNA was used to exclude variants arising from clonal hematopoiesis. When more than one clonal somatic mutation was identified in a patient's tumor tissue, the mutation with the highest variant allele frequency (VAF) was selected. For each patient, only one mutation identified in the tumor tissue was assessed in the plasma. ddPCR assays were designed to the selected mutations (Thermo Fisher Scientific). Before being used for ctDNA quantification, the performance of each assay was assessed as previously described.^16^ In brief, the linearity and technical sensitivity of the assays were assessed using a 6‐point dilution series of tumor DNA (4000 to 4 genome equivalents [GEs] in pool of 20 000 GEs of matched germline DNA). The included assays robustly and consistently detected tumor‐DNA down to 0.08% and showed excellent linearity (*R* ^2^ > .99) over three orders of magnitude input‐DNA.

To assess specificity and determine the assay specific limit of detection (LOD),^25^ each assay was applied to 94 control samples from healthy donors. The primer and probe sequences of the designed ddPCR assays are provided in Table S5.

### 2.9 DNA quantification by droplet digital PCR

Serial plasma DNA samples were analyzed on a QX200 ddPCR system according to the manufacturer's instructions (Bio‐Rad). Each analysis included matched tumor DNA (positive control), germline DNA (negative control) and a nontemplate control. Samples with a ctDNA VAF above the LOD and with at least three positive droplets were called positive.

### 2.10 Carcinoembryonic antigen analysis

Carcinoembryonic antigen (CEA) analysis was performed on a Cobas e601 platform (Roche), according to the manufacturer's recommendations using 500 μL serum. The threshold levels were set to 4.0 and 6.0 μg/L for nonsmokers and smokers, respectively. A person who had not smoked for 8 weeks before sample collection was considered a former smoker and thresholds levels set to 4.0 μg/L.

### 2.11 Statistical analysis

The primary outcome measure was recurrence free survival (RFS) assessed by standard radiologic criteria. RFS was measured from date of surgery to verified first radiologic recurrence (local or distant) or death as a result of CRC and was censored at last follow‐up or non‐CRC‐related death. Patients with no follow‐up were excluded from the study. Survival analysis was performed using the Kaplan‐Meier method. Cox proportional hazards regression analysis was used to assess the impact of ctDNA and CEA on RFS. Multivariable analysis was performed with clinical variables that were statistically significant in univariate analysis. The Efron method was used to handle ties in failure times. Proportional hazards assumptions were tested by covariate‐specific and global test of the Schoenfeld residuals. Analyses of multiple failure‐time data were performed by a conditional risk set model with an indeterminate radiologic finding used as time of entry and recurrence or censoring used as exit. Nonrandom associations between two categorical variables were determined by Fisher's exact test. For indeterminate Cox proportional hazards regression analysis time at risk were defined as the time from the indeterminate CT finding and until censoring or relapse. Wilcoxon rank‐sum tests were used to compare differences between groups. *P*‐values were based on two‐sided testing and differences were considered significant at *P* ≤ .05. Statistical analysis was performed using STATA IC/12.1 and R Statistical software, version 2.4 for Windows.

## 3 RESULTS

### 3.1 Patient characteristics and ctDNA analysis

Patient enrollment and study overview are presented in Figure 1. A total of 115 patients planned to receive curatively intended treatment for CRLM were recruited between May 2015 and December 2018. Subsequently, 47 patients were excluded due to synchronous other cancer, primary tumor not resected, lack of postoperative blood collection or lack of assays for analysis; leaving 68 patients for analysis (Figure 1). All 68 analyzed patients had R0 CRLM and primary tumor resections. The median patient age was 67.7 years and 60.3% patients were male. Radiologic recurrence was diagnosed for 70.6% of patients (48/68). The median time to recurrence was 6.1 months (range, 0.4‐28.3). The patients without recurrence had a median follow‐up of 19.7 months (range, 6.0‐37.1 months). Patient and clinical characteristics are shown in Table 1. Mutational profiling of the resected CRLM tissue identified clonal somatic mutations in all patients, and for each patient a ddPCR assay targeting a clonal mutation in either *APC*, *BRAF*, *KRAS*, *NRAS*, *PIK3CA* or *TP53* was established for ctDNA quantification (Table S6). In total 439 plasma samples from 68 patients were analyzed by ddPCR. The median cfDNA quantity extracted from plasma, and used in ddPCR, was 7247 GEs (range, 1436‐64 449 GEs). The ctDNA results for all 68 patients are listed in Table S6 and shown in Figures S1 and S2.

### 3.2 Preoperative detection of ctDNA and association to recurrence

The ctDNA was detected in 57 of 66 (86.4%) available preoperative samples (Table S6). By contrast, CEA was detected in only 14 of 21 patients (66.7%; Table S7). The quantitative level of preoperative ctDNA was not associated with subsequent recurrence status (Wilcoxon rank sum test, *P* = .37). Seventeen patients had a synchronous primary tumor at the time of preoperative blood collection, however their ctDNA positivity rate 88.2% (15/17) was not different from the patients where the primary tumor had previously been removed 85.7% (42/49) (Fisher's exact test, *P* = 1.0).

### 3.3 Postoperative ctDNA status and association to recurrence

**FIGURE 2:**
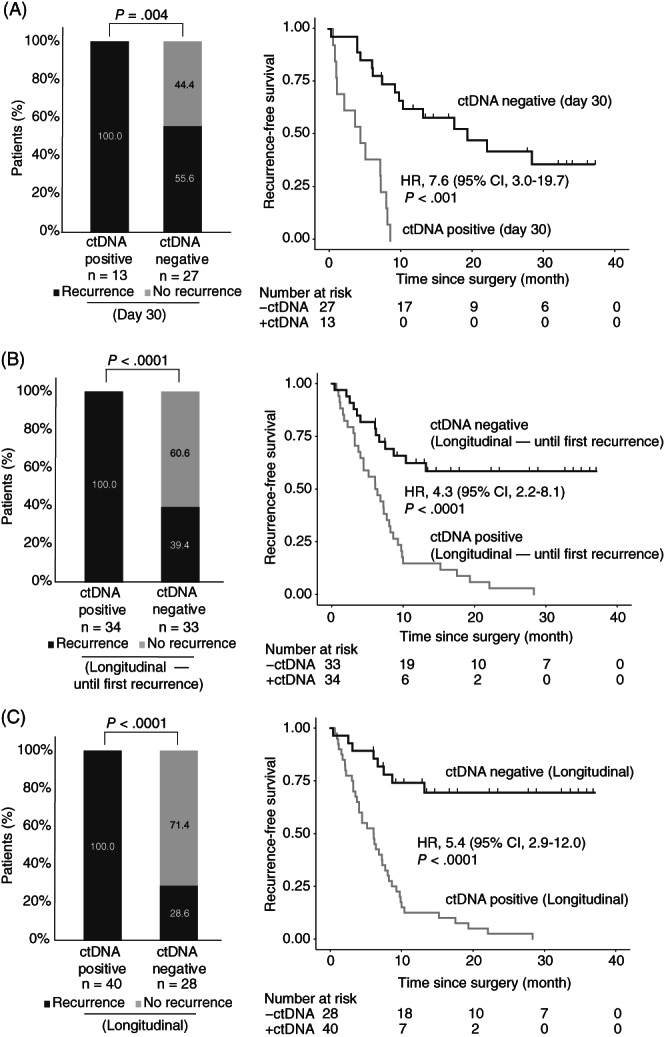
ctDNA monitoring in patients with colorectal cancer liver metastases (CRLM). (A) Kaplan‐Meier estimates (CI = 95%) of recurrence free survival for 40 CRLM patients stratified by postoperative day 30 ctDNA status. (B) Kaplan‐Meier estimates (CI = 95%) of recurrence free survival for 67 patients with longitudinal samples, stratified by longitudinal postdefinitive treatment ctDNA status before the first recurrence. (C) Kaplan‐Meier estimates (CI = 95%) of recurrence free survival for 68 patients with longitudinal samples, stratified by longitudinal postdefinitive treatment ctDNA status, all samples. A patient was called positive if one or more plasma samples within the given timeframe were ctDNA positive

Plasma collected at day 30 after surgery and prior to the start of adjuvant chemotherapy was available for 40 patients. Of these patients, 27 (67.5%) were ctDNA negative and 13 (32.5%) ctDNA positive. The recurrence rate of the ctDNA positive patients was significantly higher (100% [13 of 13 patients]; 95% CI, 77.2%‐100%) compared to those who were ctDNA negative (55.6%, [15 of 27 patients]; 95% CI, 37.3%‐72.4%; Fisher's exact test, *P* = .004; Figure 2A). The presence of ctDNA was associated with a markedly reduced RFS as compared to ctDNA negative patients (HR, 7.6; 95% CI, 3.0‐19.7; *P* < .0001; Figure 2A). Univariate analysis identified the postoperative ctDNA status at day 30 (*P* < .0001) and primary tumor N stage (*P* = .023) as significant prognostic factors. In a multivariable Cox regression model, including only the significant prognostic factors, ctDNA status was the only significant prognostic factor (*P* < .0001; Table S8).

### 3.4 Longitudinal ctDNA measurements and association to recurrence

Samples collected longitudinally from end of definitive treatment (surgery or surgery/ACT) and until diagnosis of recurrence was available from 67 patients. Longitudinal ctDNA analysis until recurrence detected ctDNA in 34 patients, and 100% (34/34, 95% CI, 89.1%‐100%) of these experienced relapse compared to only 39.4% of the ctDNA negative (13/33, 95% CI, 23.4%‐57.8%; Fisher's exact test, *P* < .0001). The serial ctDNA analysis detected relapse with 72.3% (34/47) sensitivity and 100% (20/20) specificity. The 2 year RFS was 2.9% (1 of 34 patients) for the ctDNA positive patients and 24.2% (8 of 33 patients) for the ctDNA negative (Figure 2B; HR, 4.3; 95% CI, 2.2‐8.1; *P* < .0001). In a multivariable Cox regression model, including only the significant prognostic factors both primary tumor N stage and ctDNA status were significant prognostic factor (*P* < .05; Table S8).

**FIGURE 3:**
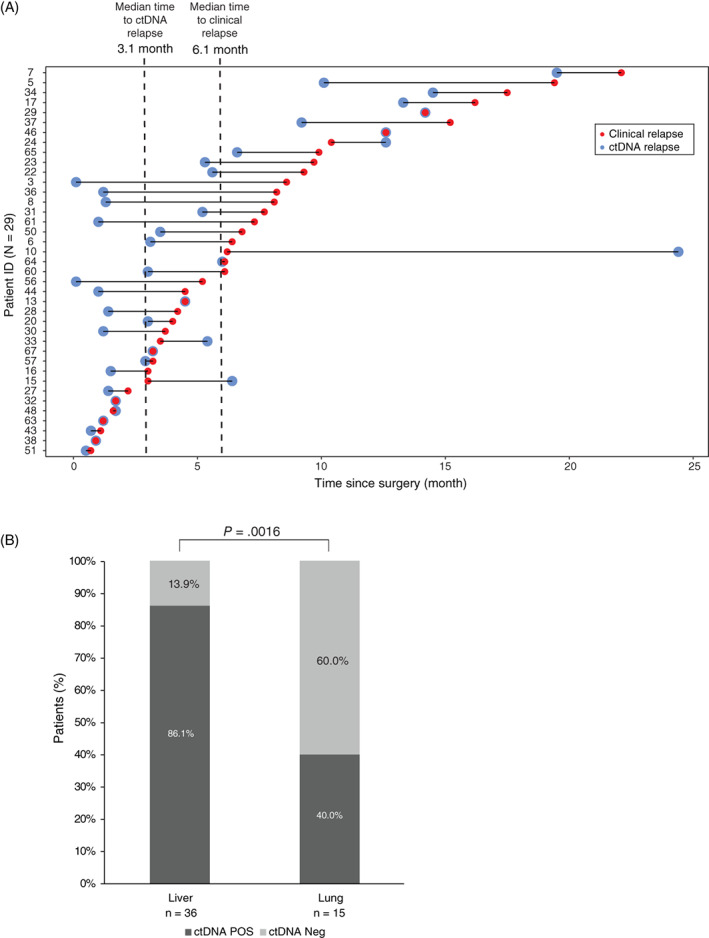
Associations between ctDNA relapse and clinical relapse and between ctDNA status and recurrence location at time of relapse. (A) Patients are sorted by time to recurrence. Only patients with blood drawn prior to or at the day of CT imaging are included (N = 39). Patient ID 55 was excluded because the first longitudinal blood sample was drawn 2.8 months subsequent to the relapse. (B) ctDNA positivity of matched blood samples from patients with liver or lung metastases were unevenly distributed (Fisher's exact test, *P* = .0016)

Extending the longitudinal analysis to also include samples collected after first recurrence, increased sensitivity to 83.3% (40/48) while specificity remained at 100% (20/20). The recurrence rate of the ctDNA positive patients was significantly higher (100% [40 of 40 patients]; 95% CI, 87.4%‐100%) than for the ctDNA negative (28.6%, [8 of 28 patients]; 95% CI, 14.0%‐48.9%; Fisher's exact test, *P* < .0001; Figure 2C). In a multivariate Cox regression model, ctDNA status and primary tumor N stage were the only significant prognostic factors (*P* < .05; Table S8). Longitudinal ctDNA analysis revealed molecular residual disease up to 9.3 months before clinical recurrence was diagnosed by standard‐of‐care CT imaging. The median lead time of ctDNA compared to imaging (time to clinical recurrence—time to ctDNA recurrence) was 2.5 months (95% CI, 1.2‐3.9; Wilcoxon signed rank test; *P* = .001; Figure 3A).

### 3.5 Paired ctDNA and CEA status and association to recurrence

For both ctDNA and CEA the pre‐OP positive rate was 75% (12/16). In the postoperative setting, 27 paired ctDNA and CEA measurements were available from samples collected longitudinally from end of definitive treatment (surgery or surgery/ACT) and until diagnosis of recurrence. Comparing the sensitivities of ctDNA and CEA we found that the sensitivity for detecting recurrence during was 77.3% (17/22) and 54.5% (12/22) for ctDNA and CEA, respectively. The specificity of the ctDNA and CEA analyses were 100% (5/5) and 80% (4/5), respectively. The ctDNA positive patients had a markedly increased recurrence risk (HR, 4.0; 95% CI, 2.4‐11.3; *P* = .008) whereas no statistical association was observed between CEA and recurrence (HR, 2.0; 95% CI, 0.8‐4.9; *P* = .115).

### 3.6 ctDNA status, disease recurrence and site of recurrence

At first recurrence, the most common recurrence site was the liver 60.4% (29/48). The second most common recurrence site was the lungs 13/48 (27.1%). Synchronous relapse to both liver and lungs was observed in 4/48 (8.3%), while relapse to other sites was rare 2/48 (4.2%; Table S1). In seven of the eight patients (87.5%) where no ctDNA were detected during surveillance, the recurrences located to the lungs, and in one patient (12.5%) to the liver. Based on these observations, lung recurrences were 15.1 times more likely to be ctDNA negative compared to liver recurrences (OR, 15.1; 95% CI, 1.7‐136; *P* = .016). The mean diameter of the lung metastases was 8.1 mm (SD 2.7).

To increase the number of recurrence events, we extended the analysis to include also recurrence events occurring beyond first recurrence. By this, we reached 36 and 15 recurrence events located to the liver and lungs, respectively. All other recurrence sites were infrequent, and consequently excluded from further analysis due to lack of statistical power. Analysis of blood samples collected at the time of recurrence revealed detection of ctDNA concurrent with 86% (31/36) of the liver metastases and 40% (6/15) of the lung metastases (Fisher's exact test, *P* = .0016; Figure 3B). To investigate if the false negative observations could be related to the selected ddPCR markers, we procured tumor DNA from four lung lesions not detected by ddPCR. In all four cases, the tumor specific mutations selected as ddPCR markers were confirmed to be present in the lung metastases (Figure S3). These results indicate that the inability to detect ctDNA in the plasma was not due to the metastatic lesions not harboring the mutation, but more likely a result of the ctDNA level in these patients being below our LOD, despite the high plasma volume analyzed (8 mL).

### 3.7 Association between ctDNA status and outcome of follow‐up after indeterminate CT findings

**FIGURE 4:**
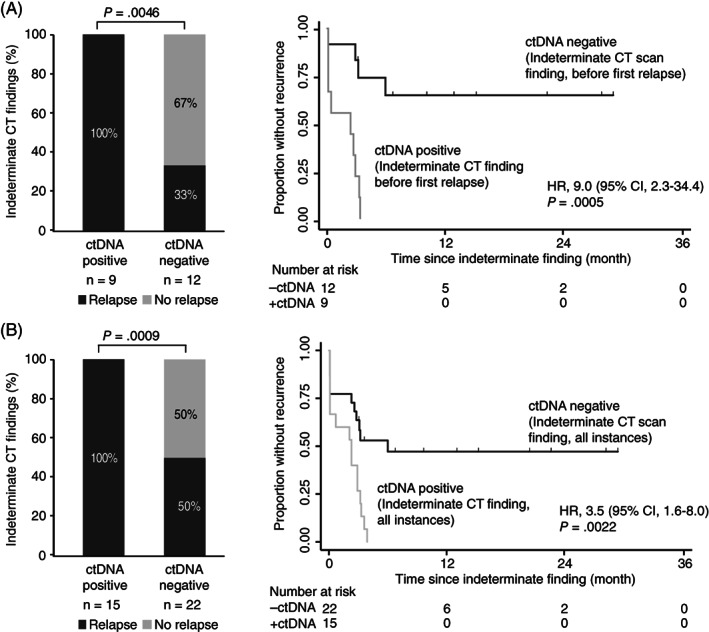
ctDNA stratified relapse rates of patients with indeterminate CT findings. (A) Kaplan‐Meier estimates (CI = 95%) of recurrence free survival for 21 CRLM patients with indeterminate findings stratified by concomitant ctDNA status before first relapse. (B) Kaplan‐Meier estimates (CI = 95%) of recurrence free survival for all patients with indeterminate findings stratified by concomitant ctDNA status. A patient was called positive if a concomitant plasma sample was ctDNA positive

Inconclusive CT scans were observed in 21/68 (30.1%) of patients during surveillance. It affected both nonrecurrence patients (N = 7) and recurrence patients (N = 14). Notably, the recurrence patients with indeterminate CT findings experienced a delay in intervention compared to the other recurrence patients. The mean time from initial CT imaging to intervention was 3.8 months (range, 3.0‐6.7 months) in patients with indeterminate findings whereas it was 1.0 month (range, 0.3‐3.3 months) in patients without such findings (Wilcoxon signed rank test; *P* < .0001). The ctDNA status at the time of the indeterminate CT scan predicted patient outcome (Figure 4A). Nine patients were ctDNA positive and 100% (9/9; PPV = 100%) of these were later diagnosed with recurrence. The remaining 12 patients were ctDNA negative and only 4/12 (33.3%; NPV = 66.7) recurred (Fisher's exact test, *P* = .0046, Figure S4). Patients that were ctDNA positive at the time‐point of an indeterminate CT finding had a significantly increased recurrence risk compared to patients that were ctDNA negative (HR, 9.0; 95% CI, 2.3‐34.4; *P* = .0005; Figure 4A).

Extending the analysis to include indeterminate findings occurring after the diagnosis of the first recurrence, 37 indeterminate CT findings with a concomitant blood sample were identified. We observed a positive predictive accuracy of 100% and a negative predictive accuracy of 50% (Fisher's exact test, *P* = .0009; Figure 4B). Patients that were ctDNA positive at the time‐point of an indeterminate CT finding had a significantly increased recurrence risk (HR, 3.5; 95% CI, 1.6‐8.0; *P* = .0022; Figure 4B).

## 4 DISCUSSION

For patients with resectable CRLM, there are currently no biomarkers with proven clinical utility for guiding the use of adjuvant chemotherapy or CT‐imaging based surveillance and decision‐making. We report here the results of a comprehensive study investigating serial ctDNA profiling before and during surveillance after curatively intended resection of CRLM. The present study demonstrates the potential impact of serial ctDNA analysis as a marker for: (1) guiding decision‐making after indeterminate CT‐findings, (2) early start of chemotherapy when there are no signs of residual or recurrent disease on CT scans and (3) imaging.

The prognostic significance of ctDNA analysis after CRLM resection in our series of patients reproduces the findings in two recent ctDNA studies of patients with resected CRLM. In these studies 83.3% (10/12)^21^ and 79.4% (27/34)^20^ of patients with detectable postoperative ctDNA had disease recurrence, whereas in the current study 100% (13/13) of these patients recurred. Patients that are ctDNA negative, by contrast appear to have a lower risk of recurrence risk with 55.6% (15/27) in the current study and 32.4% to 41.7% in the earlier studies. In the current study, just one of the postoperative ctDNA positive patients receives adjuvant chemotherapy, which may explain the higher recurrence rate compared to the other studies where clearance of ctDNA by ACT was observed in a subset of patients. These data indicate that further studies involving ACT de‐escalation strategies have to be carefully designed, due to the fact that, albeit the impact of the therapy likely is minimal, it does have an effect.

Consistent with the findings from our previous studies in early stage colon cancer^16^, ^17^, ^18^ our study confirms the possibility that serial ctDNA analysis is a potential marker for detection of minimal residual disease and early relapse detection even in a high intensity surveillance setting where CT imaging are very frequent.^9^

In our study, 100% (40/40) patients positive for ctDNA after surgery and ACT relapsed, which is very much in accordance with the 100% (11/11) observed by Tie et al^21^ and to a lesser extent with the 77.3% (17/22) presented by Wang et al.^20^ The apparent discrepancy between the studies by Tie et al and our study and the study by Wang et al might be explained by less follow‐up in the latter study or might be due to the lack of consensus with regard to ctDNA threshold.^20^

Longitudinal ctDNA plasma analyses in CRC cohorts have previously shown that ctDNA was detected 8.7 to 10 months ahead of radiological recurrence.^16^, ^17^, ^18^ In the current study, we found that ctDNA detected recurrence with median lead time of 2.5 months compared to CT imaging. This finding in a high‐risk cohort with standard of care CT imaging every 3 months show that ctDNA analyses have potential to improve the postoperative management of patients treated for CRLM.

We found that the ability to detect ctDNA postoperatively was associated with the metastatic site. Lung metastases were 15.1 times more likely to be ctDNA negative than single liver metastases (*P* = .0004). Most probable, this discrepancy is an effect of better radiographic resolution in the lung than in the liver, resulting in lung metastases being smaller when detected. The mean diameter of lung metastases in our study were 8.1 mm, which according recent studies, is below the 10 mm threshold needed for lung metastases to shed sufficient tumor DNA fragments to allow detection by KRAS ddPCR or targeted deep sequencing.^20^, ^26^, ^27^ An alternative explanation could be that the marker mutation selected for ctDNA detection was subclonal in the primary tumor, and not present in the relapsing cells. However, analysis of lung metastasis biopsies from four ctDNA negative cases, confirmed that this was not the case. Hence, low shedding from the small lung lesions is the most likely explanation, suggesting that a larger plasma volume than the 8 mL used in our study, is needed to sample sufficient tumor DNA from small lung metastases cases. At least when using a single ctDNA marker ddPCR approach, as done here. Potentially, ctDNA‐detection methods employing multiple ctDNA markers may enable robust detection. Additional markers increase the likelihood of the plasma sample containing sufficient tumor DNA fragments to enable detection. The additional markers could be tumor specific single nucleotide and copy number variants, and cfDNA‐fragment lengths. Recently, we reported how cfDNA fragment length analysis also can provide a ctDNA discriminatory signal.^28^ The findings suggest that until a ctDNA approach with improved sensitivity for detection of lung metastases is identified, radiographic imaging of the lungs should remain a central modality in CRLM surveillance.

Postoperative surveillance after CRC and CRLM resection include frequent CT imaging and clinical challenge is the frequent occurrence of indeterminate CT findings.^11^, ^12^ In the present study, nearly one third (31%) of the 68 patients eligible for ctDNA analyses had one or more indeterminate findings. In agreement with previous reports, the consequence was additional radiographic work‐up and a delay in diagnosis and intervention for the patients who eventually were diagnosed with recurrence.^12^ The consequence of the delay may be an increased tumor burden and a reduced efficacy of the intervention. This line of thinking is supported by a recent study, where serial ctDNA measurements were used to estimate the growth of colorectal metastases. Growth was extensive ranging from 25% to 143% per month.^29^ Consequently, it most likely would be a benefit for the patients with indeterminate findings if the delay to intervention could be avoided or minimized. To address this, we explored if a ctDNA assessment already at the time of the indeterminate finding could identify the subset of patients who would later be diagnosed with relapse. ctDNA was detected in 42% (9/21) of patients with indeterminate CT findings. All ctDNA positive patients recurred, indicating a PPV = 100%. The hazard ratio associated with being ctDNA positive was 9.0 (95% CI, 2.3‐34.4; *P* = .0005). The sensitivity for detecting recurrence at the time‐point of indeterminant CT‐findings was 64% (9/14). To the best of our knowledge, this is the first demonstration of the potential value of ctDNA for guiding decision‐making at indeterminant findings. It opens a new venue for earlier intervention for the large fraction of CRLM patients, whose initial CT‐finding is inconclusive.

There are several limitations to our study, including a limited sample size from a single hospital and the potential risk for false positive findings related to subset analyses. Furthermore, the lead‐time of ctDNA compared radiographic imaging for detection of recurrence may be an overestimate as blood sampling was slightly more frequent than imaging. Moreover, while our tumor‐informed single marker ctDNA detection strategy is highly specific in detecting molecular residual disease, there remains a window of opportunity for improving the analytical sensitivity. The sensitivity of our serial analysis was 83.3%, that is, in 8 of 48 recurrence patients our approach was unable to detect ctDNA. A promising approach for increasing sensitivity is to increase the number of ctDNA markers, though even with improved ctDNA detection strategies, false negative results can still occur due to low shedding tumors. Moreover, as we have exemplified for lung metastases, other modalities may in some instances have better sensitivities than ctDNA. Location of the false negative results and the sample size was limited. Ultimately, the optimal surveillance approach will have to balance sensitivity, specificity, cost and throughput.

In summary, we have added to the growing evidence of clinical value of serial assessment of ctDNA during follow‐up after curatively intended surgery of CRLM. We have confirmed the prognostic power of ctDNA both at defined time points and in serial analysis, and for the first time, demonstrated the potential clinical value of ctDNA for guiding clinical decision making at indeterminate CT findings. Further prospective studies, where the ctDNA results are used to inform patient management are required to assess the clinical utility of ctDNA‐guided approaches for CRLM surveillance.

## CONFLICT OF INTEREST

The authors declare no conflict of interests.

## AUTHOR CONTRIBUTIONS

Conception and design: Thomas Reinert and Claus Lindbjerg Andersen. Development of methodology: Thomas Reinert, Tenna Vesterman Henriksen and Claus Lindbjerg Andersen. Acquisition of data (provided animals, acquired and managed patients, provided facilities, etc): Thomas Reinert, Lena Marie Skindhøj Petersen, Tenna Vesterman Henriksen, Marie Øbo Larsen, Søren Rasmus Palmelund Krag, Anders Riegels Knudsen, Frank Viborg Mortensen, Claus Lindbjerg Andersen. Analysis and interpretation of data (eg, statistical analysis, biostatistics, computational analysis): Thomas Reinert, Lena Marie Skindhøj Petersen, Tenna Vesterman Henriksen, Mads Heilskov Rasmussen, Amanda Frydendahl Boll Johansen, Michael Knudsen, Iver Nordentoft, Søren Vang, Søren Rasmus Palmelund Krag, Anders Riegels Knudsen, Frank Viborg Mortensen, Claus Lindbjerg Andersen. Writing, review and/or revision of the article: Thomas Reinert, Lena Marie Skindhøj Petersen, Tenna Vesterman Henriksen, Marie Øbo Larsen, Mads Heilskov Rasmussen, Amanda Frydendahl Boll Johansen, Nadia Øgaard, Michael Knudsen, Iver Nordentoft, Søren Vang, Søren Rasmus Palmelund Krag, Anders Riegels Knudsen, Frank Viborg Mortensen, Claus Lindbjerg Andersen. Administrative, technical or material support (ie, reporting or organizing data, constructing databases): Thomas Reinert, Lena Marie Skindhøj Petersen, Tenna Vesterman Henriksen, Anders Riegels Knudsen, Frank Viborg Mortensen, Claus Lindbjerg Andersen. Study supervision: Claus Lindbjerg Andersen.

## ETHICS STATEMENT

The study was approved by the Committees on Biomedical Research Ethics in the Central Region of Denmark (1‐16‐02‐453‐14) and was performed in accordance with the Declaration of Helsinki. All participants provided written informed consent.

## Supporting information


**Appendix S1** Supporting Information.

**Table S1** Clinicopathological patients characteristics
**Table S2**. Summary of tissue samples and ctDNA biomarker identification.
**Table S3**. Sequencing coverage and quality statistics for each sample.
**Table S4**. Regions captured by the duplex capture panel. Designed based on data from TCGA data of CRC patients and it is expected to identify at least one mutation in 99% of CRC and CRLM patients. The panel includes some of the most commonly mutated
**Table S5**. Primer and probe sequences and amplification protocols for ddPCR assays.
**Table S6**. ctDNA results for all 439 plasma samples analyzed by ddPCR.
**Table S7**. CEA results for all 149 samples
**Table S8**. Relapse‐free survival analyses by clinicopathological variables, ctDNA status and CEA after treatment. Analyses are performed at day 30, longitudinal—until first recurrence and longitudinal.

## Data Availability

The data that support the findings of our study are available from the corresponding author upon reasonable request.
