# Supplementary material for: Circulating tumor DNA for prognosis assessment and postoperative management after curative‐intent resection of colorectal liver metastases
Source: Int J Cancer. 2022 Jan 19;150(9):1537–48. doi: 10.1002/ijc.33924 (PMC9303459; doi:10.1002/ijc.33924)

Circulating tumor DNA for prognosis assessment and postoperative management after curative-intent resection of colorectal liver metastases

Thomas Reinert(PhD)*, Lena Marie Skindhøj Petersen(MSc)*, Tenna Vesterman Henriksen(MSc), Marie Øbo Larsen(MD), Mads Heilskov Rasmussen(PhD), Amanda Frydendahl Boll Johansen(MSc), Nadia Øgaard(MSc), Michael Knudsen(PhD), Iver Nordentoft(PhD), Søren Vang(PhD), Søren Rasmus Palmelund Krag(MD), Anders Riegels Knudsen(MD), Frank Viborg Mortensen(MD), Claus Lindbjerg Andersen(PhD)**

*These authors contributed equally.

**Corresponding author.

Table of content (Supplementary content)

Supplementary Tabels S1 to S8 (Available in separate Excel file)
Supplementary Figure S1
Supplementary Figure S2
Supplementary Figure S3
Supplementary Figure S4

Suppl. Figure S1. Detailed ctDNA results and disease course information.

Detailed disease courses, applied treatment, and longitudinal ctDNA analyses for all 68 patients. ctDNA is represented as copies/mL plasma.

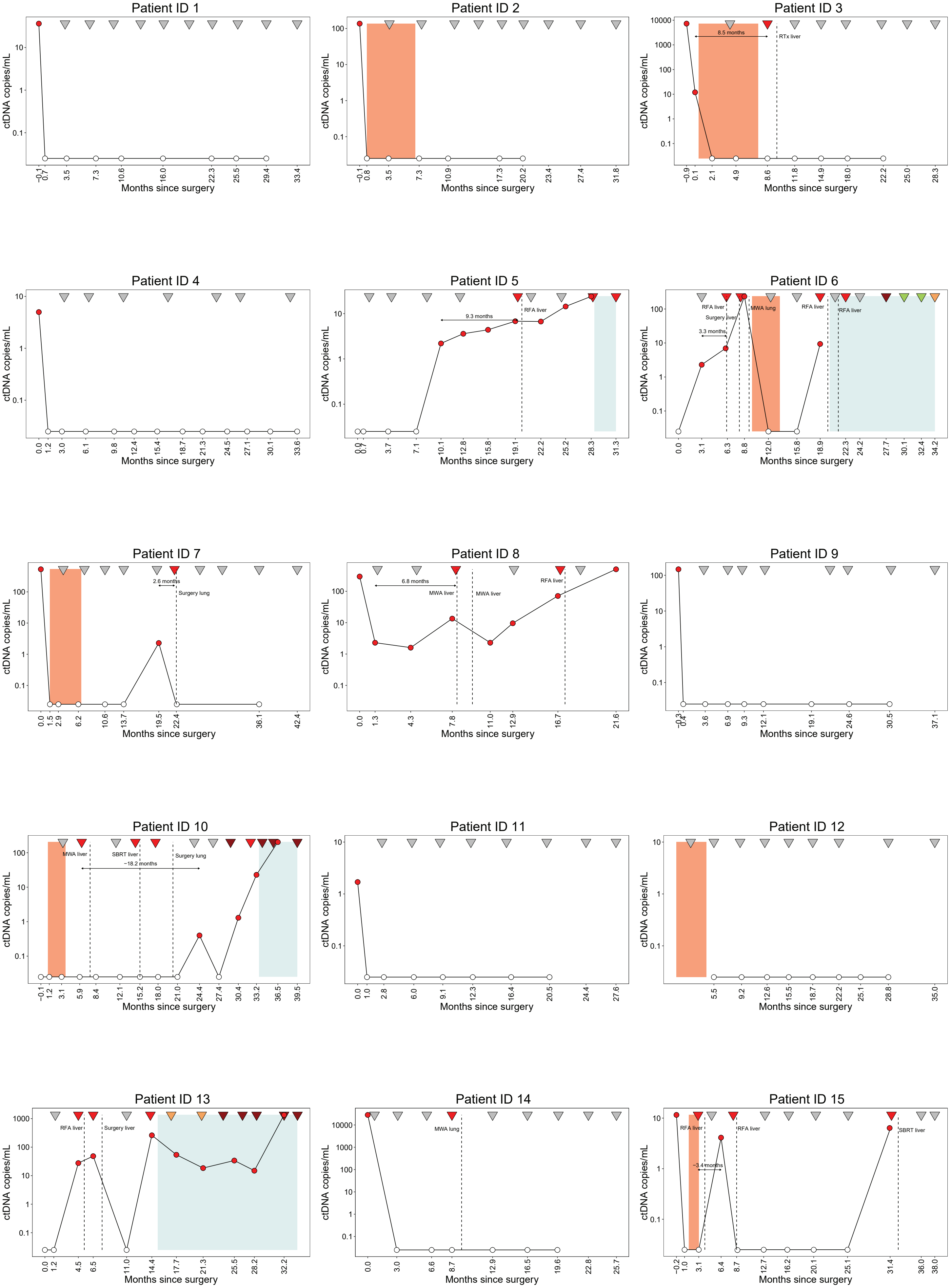

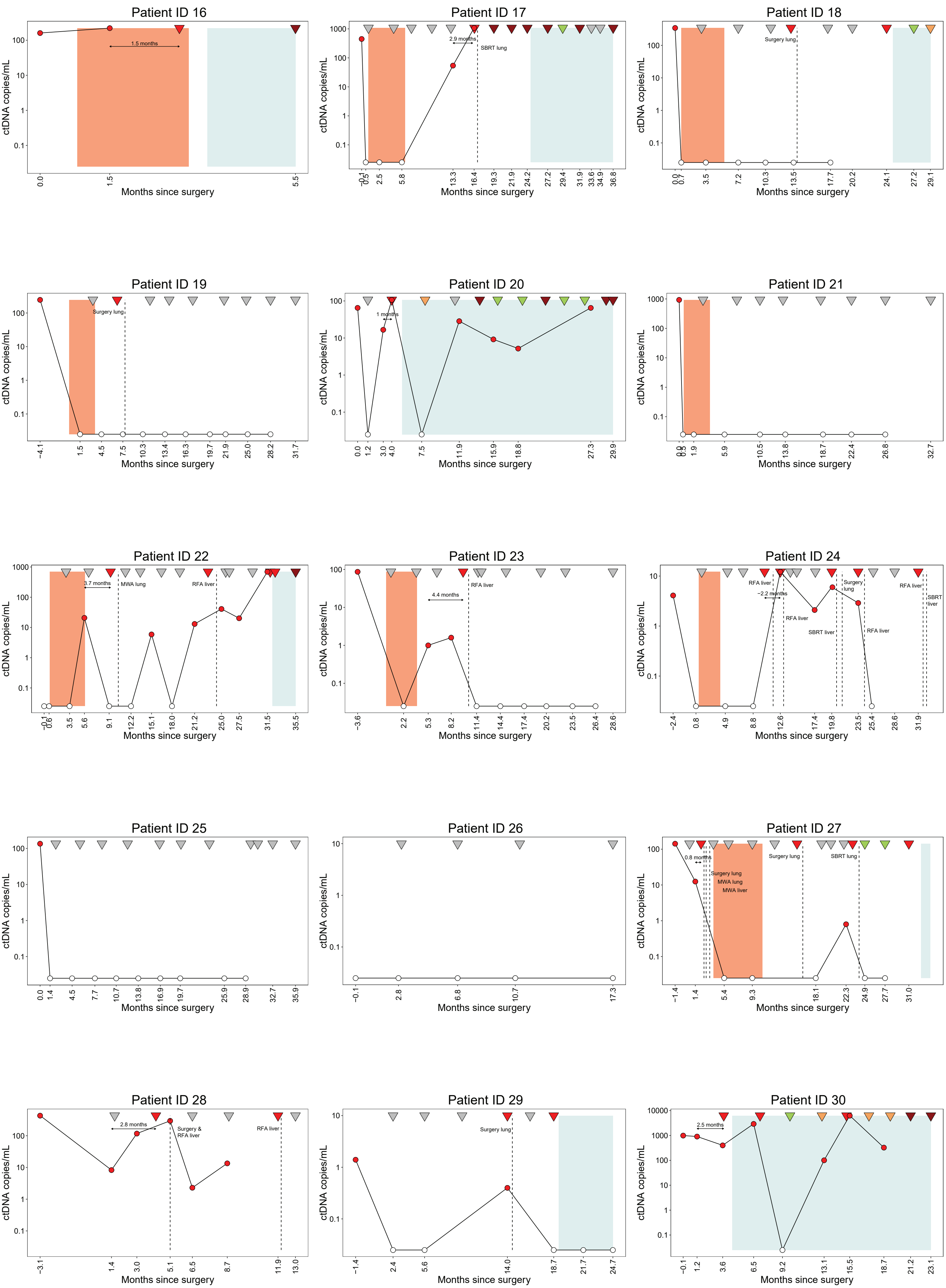

Patient ID 16
ctDNA copies/mL
1.5 months
0.0
1.5
5.5
Months since surgery
Patient ID 17
ctDNA copies/mL
2.9 months
SBRT lung
−0.1
0.5
2.5
5.8
13.3
16.4
19.3
21.9
24.2
27.2
29.4
31.9
33.6
34.9
36.8
Months since surgery
Patient ID 18
ctDNA copies/mL
Surgery lung
0.0
0.7
3.5
7.2
10.3
13.5
17.7
20.2
24.1
27.2
29.1
Months since surgery
Patient ID 19
ctDNA copies/mL
Surgery lung
−4.1
1.5
4.5
7.5
10.3
13.4
16.3
19.7
21.9
25.0
28.2
31.7
Months since surgery
Patient ID 20
ctDNA copies/mL
1 months
0.0
1.2
3.0
4.0
7.5
11.9
15.9
18.8
27.3
29.9
Months since surgery
Patient ID 21
ctDNA copies/mL
0.0
0.5
1.9
5.9
10.5
13.8
18.7
22.4
26.8
32.7
Months since surgery
Patient ID 22
ctDNA copies/mL
3.7 months
MWA lung
RFA liver
−0.1
0.6
3.5
5.6
9.1
12.2
15.1
18.0
21.2
25.0
27.5
31.5
35.5
Months since surgery
Patient ID 23
ctDNA copies/mL
4.4 months
RFA liver
−3.6
2.2
5.3
8.2
11.4
14.4
17.4
20.2
23.5
26.4
28.6
Months since surgery
Patient ID 24
ctDNA copies/mL
RFA liver
~2.2 months
RFA liver
SBRT liver
Surgery lung
RFA liver
RFA liver
SBRT liver
−2.4
0.8
4.9
8.8
12.6
17.4
19.8
23.5
25.4
28.6
31.9
Months since surgery
Patient ID 25
ctDNA copies/mL
0.0
1.4
4.5
7.7
10.7
13.8
16.9
19.7
25.9
28.9
32.7
35.9
Months since surgery
Patient ID 26
ctDNA copies/mL
−0.1
2.8
6.8
10.7
17.3
Months since surgery
Patient ID 27
ctDNA copies/mL
0.8 months
Surgery lung
MWA lung
MWA liver
Surgery lung
SBRT lung
−1.4
1.4
5.4
9.3
18.1
22.3
24.9
27.7
31.0
Months since surgery
Patient ID 28
ctDNA copies/mL
2.8 months
Surgery & RFA liver
RFA liver
−3.1
1.4
3.0
5.1
6.5
8.7
11.9
13.0
Months since surgery
Patient ID 29
ctDNA copies/mL
Surgery lung
−1.4
2.4
5.6
14.0
18.7
21.7
24.7
Months since surgery
Patient ID 30
ctDNA copies/mL
2.5 months
−0.1
1.2
3.6
6.5
9.2
13.1
15.5
18.7
21.2
23.1
Months since surgery

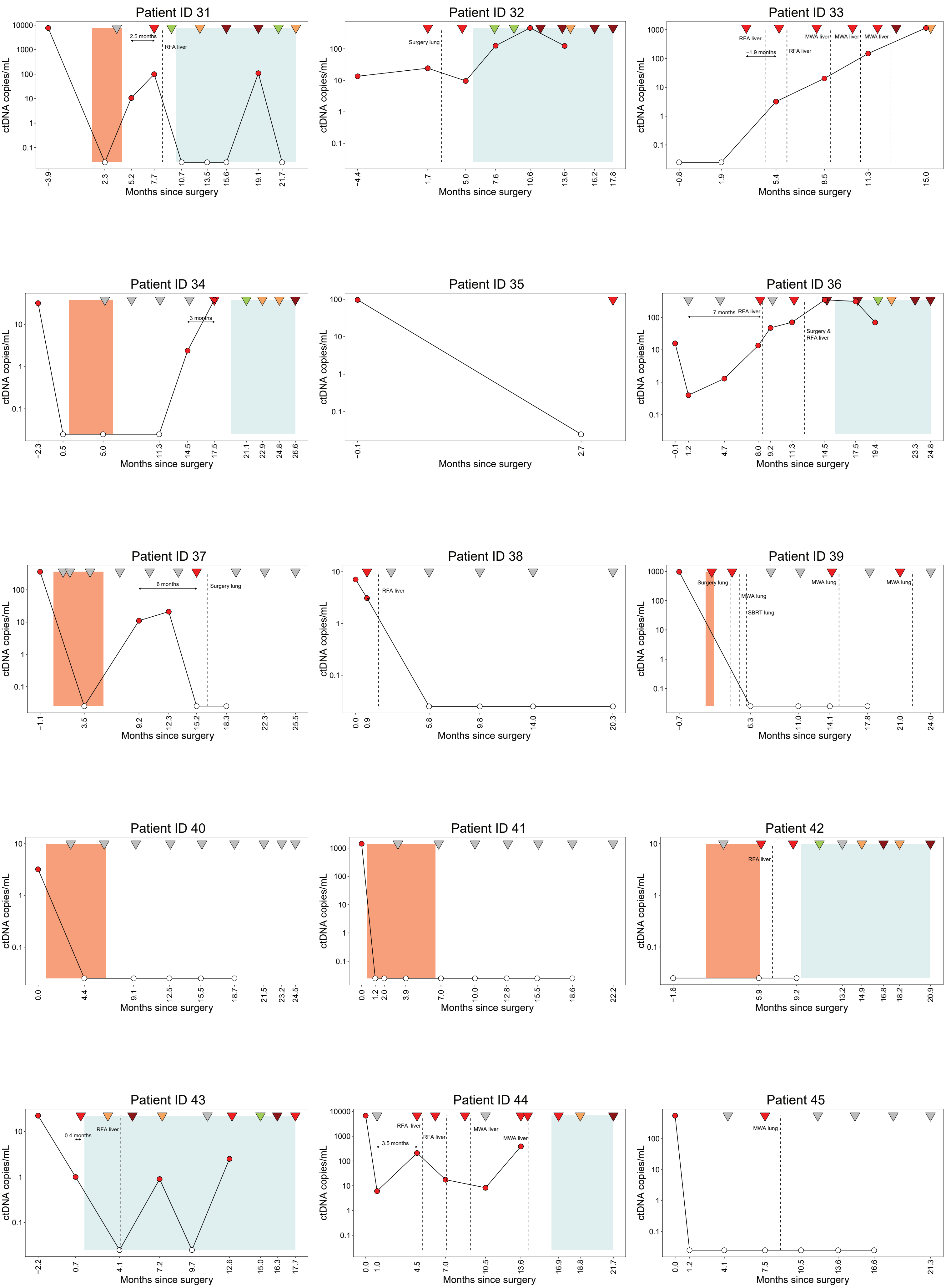
Patient ID 31
2.5 months
RFA liver
Patient ID 32
Surgery lung
Patient ID 33
RFA liver
~1.9 months
RFA liver
MWA liver
MWA liver
MWA liver
Patient ID 34
3 months
Patient ID 35
Patient ID 36
7 months
RFA liver
Surgery &
RFA liver
Patient ID 37
6 months
Surgery lung
Patient ID 38
RFA liver
Patient ID 39
Surgery lung
MWA lung
SBRT lung
MWA lung
MWA lung
Patient ID 40
Patient ID 41
Patient 42
RFA liver
Patient ID 43
0.4 months
RFA liver
Patient ID 44
RFA liver
3.5 months
RFA liver
MWA liver
MWA liver
Patient 45
MWA lung
ctDNA copies/mL
Months since surgery

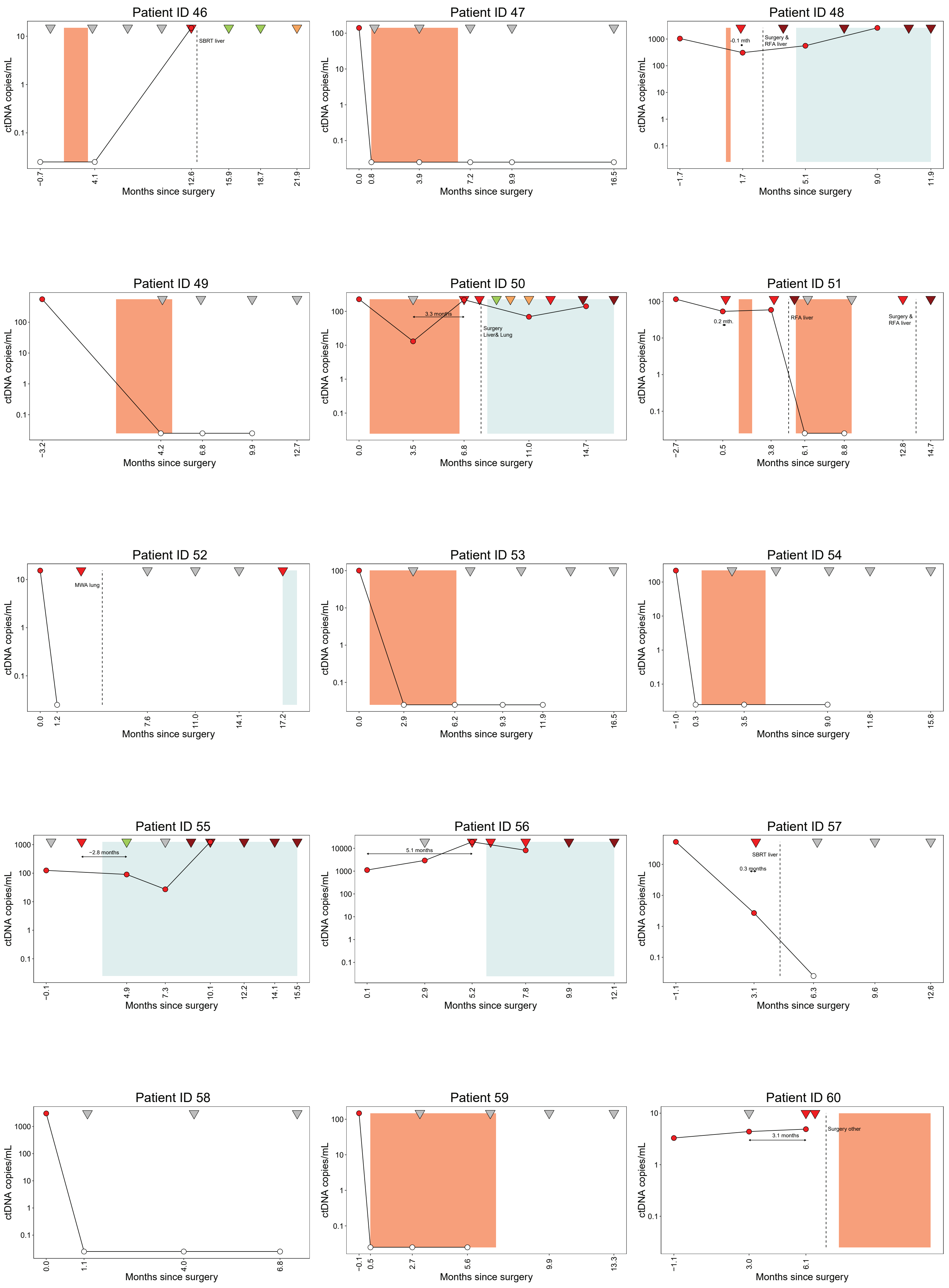

Patient ID 46
SBRT liver
Patient ID 47
Patient ID 48
-0.1 mth
Surgery &
RFA liver
Patient ID 49
Patient ID 50
3.3 months
Surgery
Liver& Lung
Patient ID 51
0.2 mth.
RFA liver
Surgery &
RFA liver
Patient ID 52
MWA lung
Patient ID 53
Patient ID 54
Patient ID 55
−2.8 months
Patient ID 56
5.1 months
Patient ID 57
SBRT liver
0.3 months
Patient ID 58
Patient 59
Patient ID 60
3.1 months
Surgery other
ctDNA copies/mL
Months since surgery

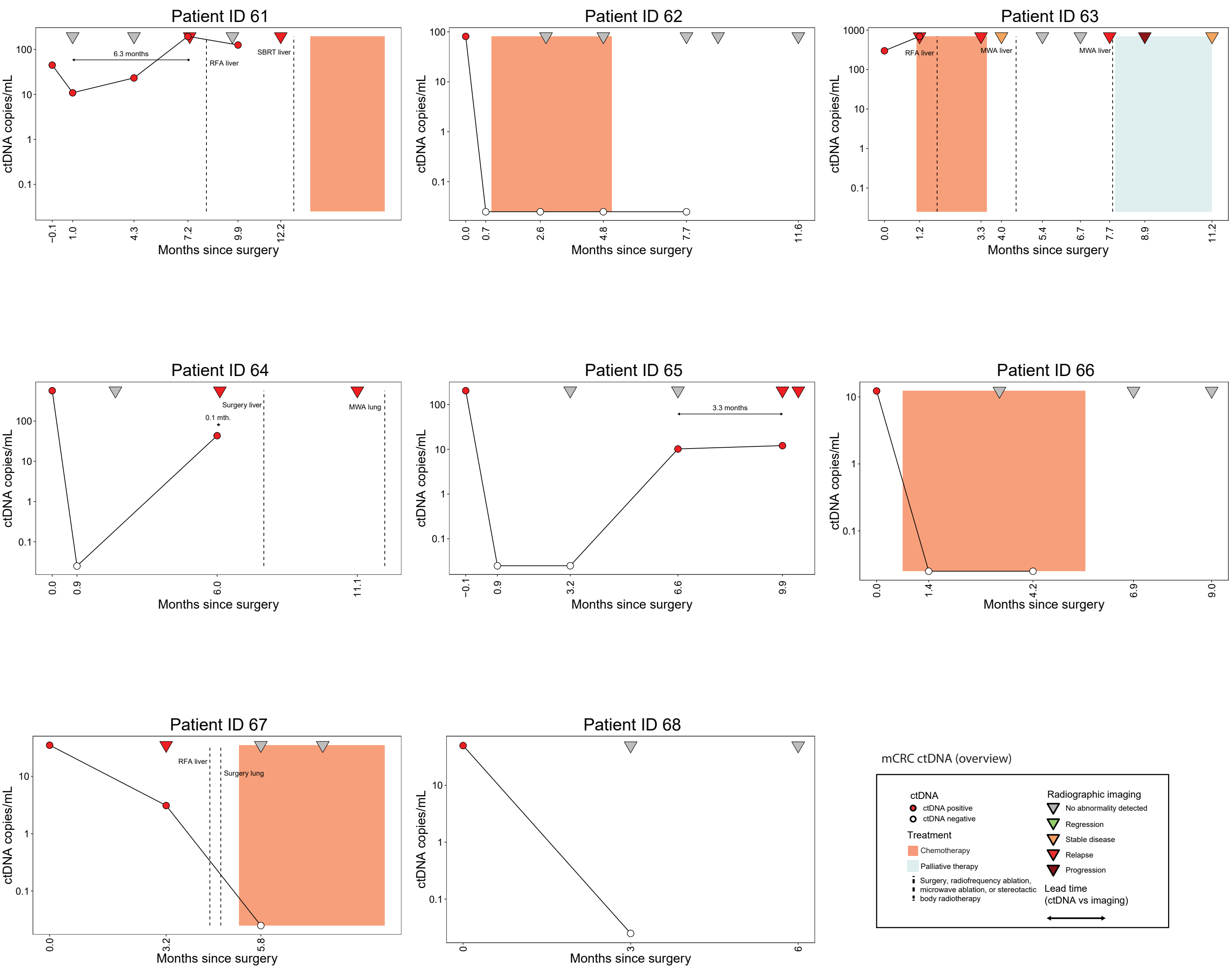

Patient ID 61
6.3 months
RFA liver
SBRT liver
ctDNA copies/mL
Months since surgery
Patient ID 62
Patient ID 63
RFA liver
MWA liver
MWA liver
Patient ID 64
Surgery liver
0.1 mth.
MWA lung
Patient ID 65
3.3 months
Patient ID 66
Patient ID 67
RFA liver
Surgery lung
Patient ID 68
mCRC ctDNA (overview)
ctDNA
ctDNA positive
ctDNA negative
Treatment
Chemotherapy
Palliative therapy
Surgery, radiofrequency ablation, microwave ablation, or stereotactic body radiotherapy
Radiographic imaging
No abnormality detected
Regression
Stable disease
Relapse
Progression
Lead time (ctDNA vs imaging)

Suppl. Figure S2. ctDNA profiling results for all patients analyzed during the study. Patients are ordered by recurrence status. Patients were considered positive if one or more plasma samples postdefinitive treatment was ctDNA-positive. Generally, longitudinal analysis revealed persistent ctDNA positivity in serial samples, with ctDNA status changing only in case of clinical intervention. Patients are sorted by relapse status and follow-up length.

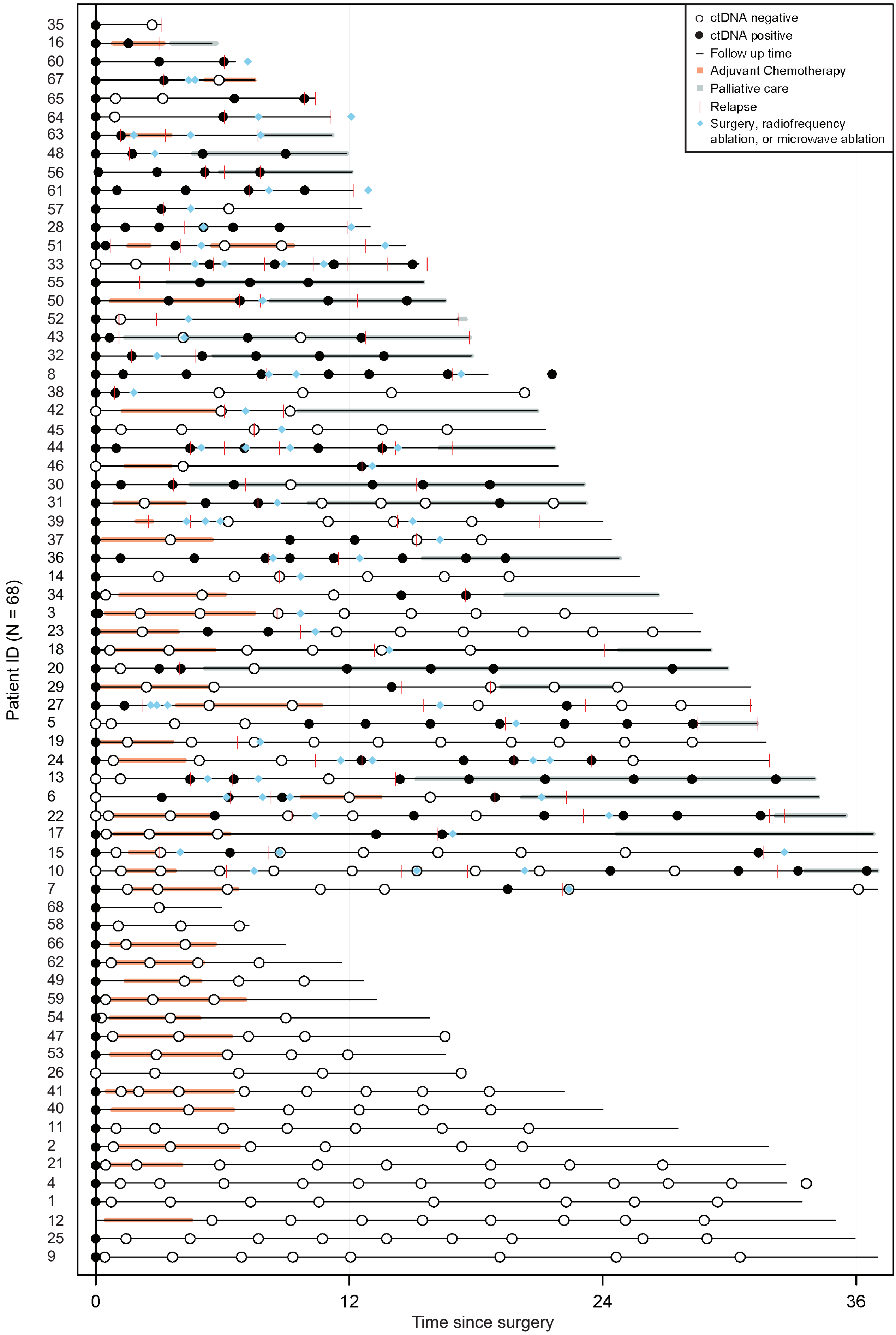

Suppl. Figure S3. Lung metastasis undetected by plasma ctDNA monitoring. Four patients that were ctDNA negative in the period up to diagnosis and surgical treatment for lung metastasis. Mutations used as ctDNA markers were identified in the primary liver metastases.

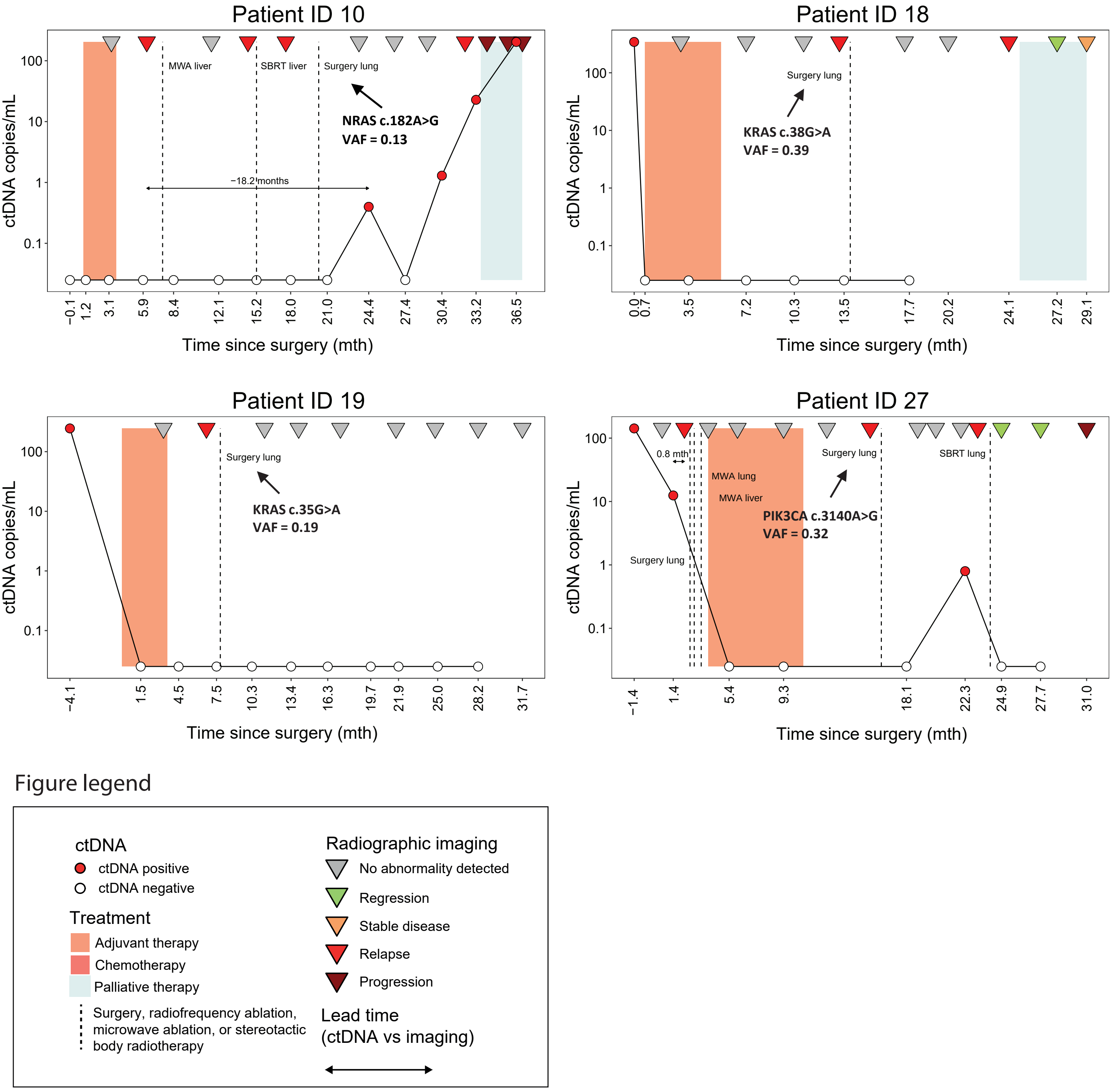

Suppl. Figure S4. Detailed ctDNA results and disease course information for patients with indeterminate radiographic imaging. Detailed disease courses for 21 patients with indeterminate radiographic imaging findings during follow up. For relapsing patients, we only include follow-up from primary treatment and until diagnosis of relapse. Shown are the results of radiographic imaging, longitudinal ctDNA analyses. Treatment periods are also indicated.

Relapse patients with indeterminate findings

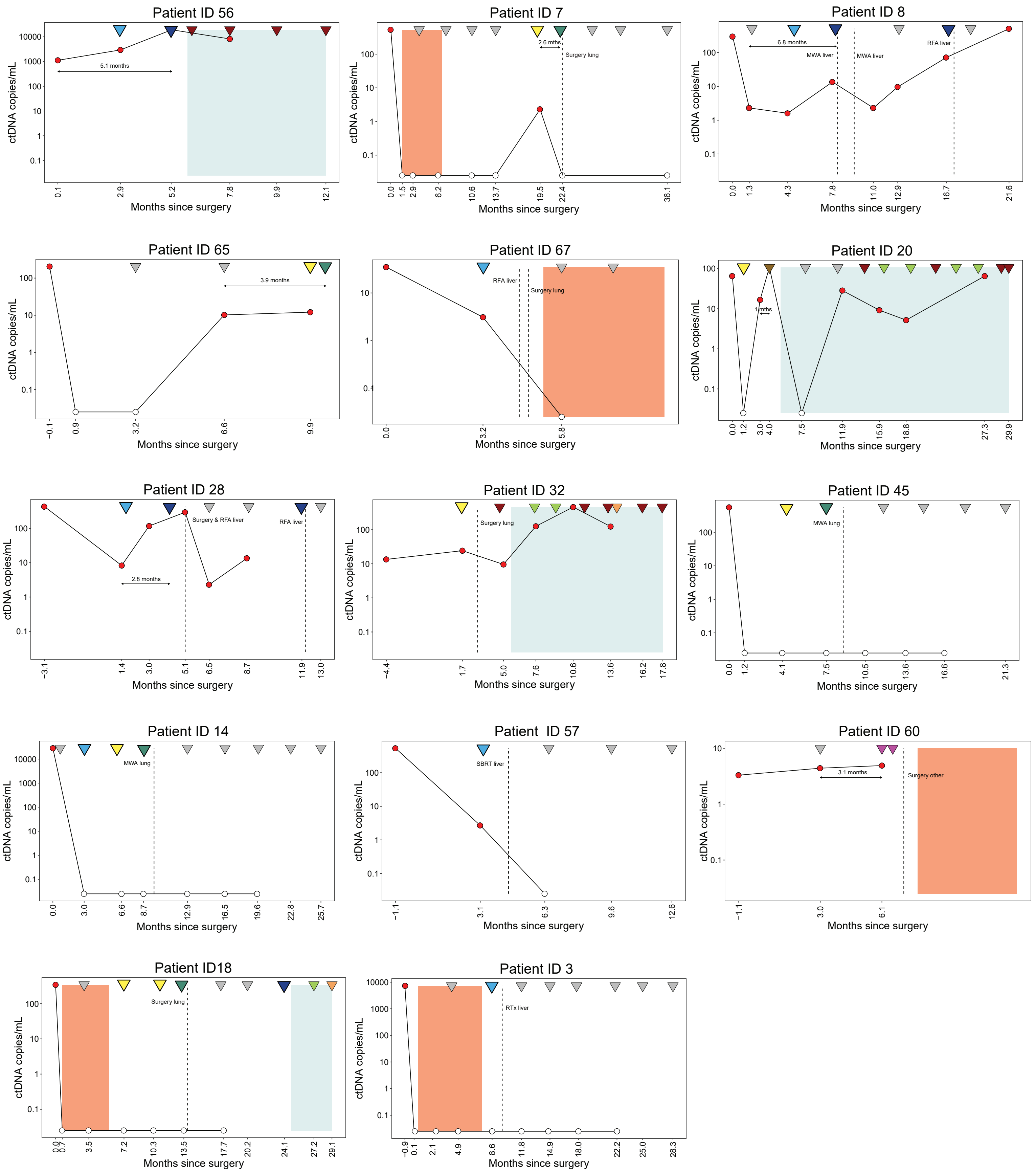

Relapse free patients with indeterminate findings

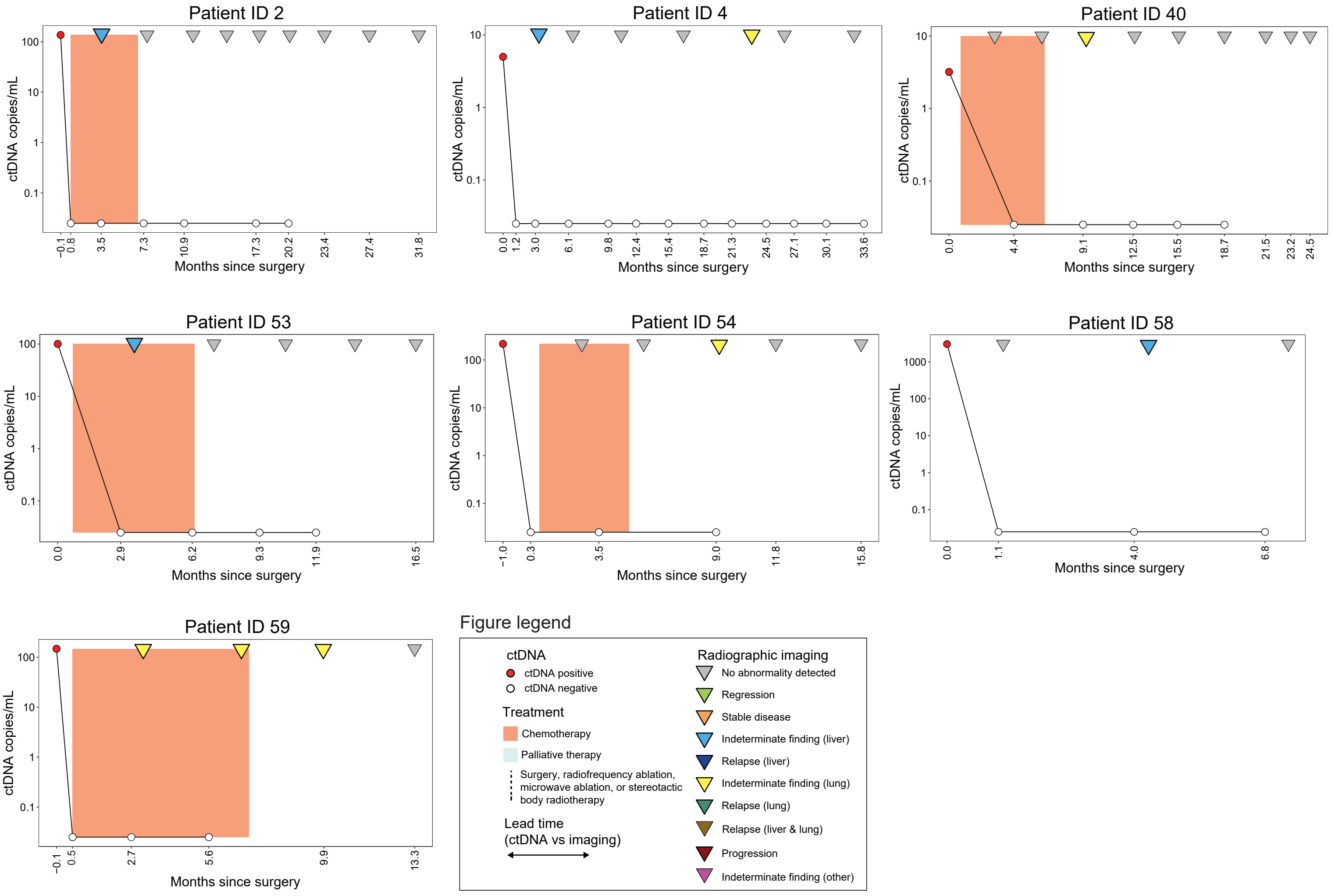

Supplement: Supplementary file 1 — Appendix S1 Supporting Information. [file IJC-150-1537-s002.pdf]
